# Evaluation of S7, a synthetic partial abscisic acid agonist, as a potential suppressor of pre-harvest sprouting in rice

**DOI:** 10.1186/s12870-026-09511-9

**Published:** 2026-07-16

**Authors:** Ju-Hee Kim, Ji-Hyeon Mun, So-Hye Jo, Jae-Kyeong Baek, Yu-Na Kim, Yeong-Seo Song, Woon-Ha Hwang, Beom-Gi Kim, Seo-Yeong Yang, Hyeon-Seok Lee

**Affiliations:** 1 Crop Production & Physiology Division, National Institute of Crop Science, Rural Development Administration, 55365, Wanju-Gun, Republic of Korea; 2 Plant Biomaterials and Biotechnology Division, Department of Agricultural Biology, National Institute of Agricultural Sciences, Rural Development Administration, Wanju, 55365 Republic of Korea; 3 Department of Crop Science, College of Agriculture and Life Science, Chungnam National University, Daejeon, 34134 Republic of Korea

**Keywords:** Pre-harvest sprouting, S7, Rice, Abscisic acid signaling, α-amylase, Seed germination

## Abstract

**Background:**

Pre-harvest sprouting (PHS) is a major constraint affecting grain quality and yield in rice, and its incidence is expected to increase with more frequent rainfall events associated with climate change. Although chemical control of PHS has largely focused on abscisic acid (ABA)-dependent regulation of seed dormancy and germination, the practical application of ABA is limited by its high cost and instability. This study investigated the potential of S7, a synthetic partial ABA agonist, as a chemical tool for suppressing PHS in rice.

**Results:**

Germination assays, controlled-environment pot experiments, molecular analyses, and field trials were conducted to evaluate the physiological and molecular responses to S7 and its agronomic applicability. S7 treatment delayed seed germination and reduced α-amylase activity, thereby suppressing PHS, without increasing endogenous ABA levels during seed imbibition. Gene expression analyses showed that S7 treatment was accompanied by ABA signaling-related transcriptional changes, including early induction of OsABI4 and reduced OsGAMYB expression, together with reduced α-amylase activity. Controlled-environment pot experiments indicated that the suppressive effect of S7 depended on application timing during grain filling, whereas field trials supported its potential effectiveness under variable phenological and grain-filling conditions.

**Conclusions:**

These findings suggest that S7-mediated suppression of germination is associated with ABA signaling-related transcriptional responses rather than increased endogenous ABA accumulation. Overall, S7 may serve as a potential non-genetic chemical tool for reducing pre-harvest sprouting in rice, although further studies are required to optimize concentration, application timing, formulation, environmental stability, and cultivar-specific responses under field conditions.

**Supplementary Information:**

The online version contains supplementary material available at https://doi.org/10.1186/s12870-026-09511-9.

## Background

Pre-harvest sprouting (PHS) refers to the premature germination of mature or nearly mature grains before harvest, leading to reduced grain quality and yield in cereal crops such as rice (*Oryza sativa* L.) and wheat (*Triticum aestivum* L.). PHS occurs when grains on the panicle or spike are exposed to environmental conditions favorable for germination, including rainfall, high humidity, and suitable temperatures [12]. Climate change is altering global precipitation patterns and increasing the frequency and intensity of extreme rainfall events in many regions. Globally, record-breaking rainfall events have increased by 12% over the past 30 years since 1981, with greater increases observed at higher temperatures [20]. In regions such as Pakistan, extreme monsoon rainfall has caused unprecedented flooding, and climate models predict that climate change may increase rainfall intensity by up to 50% [28]. Therefore, increased harvest-stage rainfall associated with climate change is expected to increase the risk of PHS in cereal production.

PHS affects the physicochemical properties of grains, including starch structure, crystallinity, and gelatinization characteristics. In rice, PHS can cause surface pitting and roughness in starch granules, reduce crystallinity, and alter grain processing quality [11]. In wheat, PHS decreases the Hagberg Falling Number, an indicator of bread-making quality, mainly because increased α-amylase activity accelerates starch degradation in the endosperm [12, 23] . PHS also reduces seed viability and market value through premature germination before harvest. Consequently, PHS can cause substantial economic losses, with annual global losses estimated to exceed USD 1 billion owing to reductions in grain quality and yield [24, 38].

Although PHS has been addressed through chemical, genetic, and breeding approaches, its occurrence is strongly influenced by environmental conditions, seed dormancy, seed-coat characteristics, α-amylase activity, and endogenous hormone balance [1, 12, 24, 30]. Among these factors, abscisic acid (ABA) is a major regulator of seed dormancy and PHS resistance because it suppresses germination-associated processes, including GA-induced α-amylase expression. ABA accumulation during seed maturation is largely controlled by biosynthetic enzymes, such as NCED, and the expression of NCED genes, including OsNCED3 in rice and NCED homologs in wheat and triticale, has been associated with higher ABA content, stronger dormancy, and increased PHS resistance [3, 6, 7]. Conversely, enhanced ABA catabolism through ABA 8′-hydroxylases or a reduced ABA/GA balance can promote dormancy release and increase PHS susceptibility [7, 33]. In addition to ABA metabolism, ABA signaling components, including the PYR/PYL–PP2C–SnRK2 module and downstream regulators such as VP1/ABI3 and ABI5, mediate seed dormancy and germination responses by modulating GA-responsive and hydrolytic enzyme pathways [32, 31, 24]. These findings indicate that PHS resistance is determined not only by endogenous ABA abundance, but also by ABA catabolism and signaling sensitivity.

However, the broader agricultural application of ABA is restricted by its high cost and instability [10, 22]. Therefore, several chemical inhibitors have been evaluated as alternatives for suppressing PHS. For example, eugenol delays rice seed germination and reduces PHS mainly by increasing endogenous ABA levels through altered expression of ABA metabolic genes, including increased OsNCED2 and reduced OsABA8OH2 expression [10]. Similarly, combined treatment with maleic hydrazide and coumarin suppresses rice seed germination by increasing ABA accumulation, modifying ROS homeostasis, and reducing α-amylase activity and starch degradation [34], while a MeJA analog delays wheat PHS by increasing ABA content, decreasing GA content, and reducing α-amylase activity [5]. These studies indicate that previously reported chemical suppressors largely act through ABA accumulation, ABA/GA hormonal balance, ROS regulation, or downstream inhibition of starch degradation. In contrast, chemical approaches that may suppress PHS through abscisic acid signaling-related responses without directly increasing endogenous abscisic acid accumulation remain insufficiently explored.

S7, a synthetic partial ABA agonist that modulates ABA signaling in plants, was identified through an in vivo screening system designed to isolate chemicals capable of inducing ABA signaling in rice protoplasts [26]. Min et al. [26] reported that treatment with 50 μM S7 promoted stomatal closure without suppressing plant growth, thereby reducing water consumption and water loss. These findings suggest that S7 can selectively activate ABA-responsive physiological outputs without fully mimicking all the effects of ABA. Given the central role of ABA in seed dormancy and germination repression, S7 may also influence germination-associated ABA signaling and thereby affect PHS. However, whether S7 can suppress PHS in rice and how it affects physiological and molecular responses during germination remain unclear.

This study investigated the potential of S7 as a chemical tool for suppressing PHS in rice. Specifically, we evaluated the effects of S7 concentration and application timing on seed germination, PHS occurrence, α-amylase activity, endogenous ABA content, ABA-related gene expression, and basic post-harvest seed performance. We hypothesized that S7 delays germination and suppresses PHS by modulating ABA signaling-related responses rather than by directly increasing endogenous ABA accumulation. We further examined the effectiveness of S7 under controlled-environment and field conditions to assess its potential applicability for PHS management in rice. By identifying effective treatment conditions for S7, this study provides a physiological and agronomic basis for developing chemical strategies to mitigate PHS risk under increasingly variable rainfall conditions.

## Materials and methods

### Ethics statement

The authors declare that all plant materials used in this study were handled in accordance with relevant institutional guidelines and regulations. The rice cultivars ‘Samkwang’, used in the germination assay and controlled-environment pot experiment, and ‘Baromi2’, used in the field experiment, were provided by Seo-Yeong Yang from the Crop Production and Physiology Division, National Institute of Crop Science, Rural Development Administration, Republic of Korea. S7 was provided by Beom-Gi Kim from the Plant Biomaterials and Biotechnology Division, Department of Agricultural Biology, National Institute of Agricultural Sciences, Rural Development Administration, Republic of Korea. Permission for the use of the plant materials was obtained from the relevant institutes.

(https://www.nics.go.kr/api/spices.do?m=100000128&homepageSeCode=nics).

### Experimental site and materials

The experiments were conducted at the National Institute of Crop Science, Jeonju, Republic of Korea (35°49ʹ19ʺ N, 127°8ʹ56ʺ E). The seed germination assay and pot experiment were conducted in controlled-environment facilities manufactured by ENT, Inc. (Bucheon, Republic of Korea), where light intensity, temperature, photoperiod, and relative humidity were regulated (Figure S1). The field experiment was conducted in paddy fields at the same institute under natural environmental conditions (Figure S2).

The rice cultivar ‘Samkwang’ (*O. sativa* L. ssp. *japonica*) was used for the seed germination assay and controlled-environment pot experiment because it has been widely used in studies evaluating rice growth, and physiological responses under weather-related stress conditions, as well as in rice pre-harvest sprouting-related experiments and artificial-rainfall-based PHS evaluations [14, 16–19]. For the field experiment, the PHS-susceptible floury rice cultivar ‘Baromi2’ (*O. sativa* L.,Registration No. 9052) was used to ensure sufficient PHS occurrence under natural field conditions and to evaluate the effectiveness of S7 in a susceptible genetic background [27]. The wheat cultivar ‘Keumkang’ (*Triticum aestivum* L.) was used for the wheat seed germination assay. Accumulated temperature after heading was calculated as the sum of daily mean air temperatures after heading and expressed as °C d.

### Experiment 1: seed germination

Rice seeds were surface-sterilized by shaking in 70% ethanol for 3 min, rinsed once with distilled water, and then disinfected with 50% commercial chlorine bleach for 40 min. The sterilized seeds were rinsed at least three times with distilled water and dried on a clean bench for more than 3 h. S7 was added to Murashige and Skoog medium at concentrations of 0, 100, 250, and 500 μM. Then, 25 mL of medium was poured into plant culture dishes (SPL Life Sciences, Pocheon, Republic of Korea; Cat. No. 310103), and 50 seeds were sown per dish. Three biological replicates were used for each treatment.

Rice seeds were incubated in a germination chamber under a 14 h light/10 h dark photoperiod for 7 d. Germination was recorded at 12, 24, 36, 48, 72, 96, and 168 h after sowing. Seeds were considered germinated when the radicle length exceeded 1 mm.

To examine whether the germination-delaying effect of S7 could also be observed in another cereal crop, wheat seeds of the cultivar ‘Keumkang’ were treated with S7 at 0, 150, 300, and 450 μM. Fifty wheat seeds were used per replicate, with three biological replicates per treatment. Wheat seed germination was recorded at 12, 18, 24, 36, 48, 60, 72, and 84 h after sowing. The S7 concentration range was determined based on Min et al. [26].

### Experiment 2: pot trial under controlled conditions

The controlled-environment pot experiment was conducted in the controlled-environment facility described above. Rice plants were grown under regulated light intensity, photoperiod, and relative humidity conditions. Until heading, the temperature was maintained at a daily mean of 25 °C, with maximum and minimum temperatures of 30 °C and 20 °C, respectively. Heading date was recorded for three panicles per hill, and the mean heading date was calculated for each plant.

S7 was applied to rice plants at concentrations of 0, 250, and 500 μM at 3, 7, or 10 d before the accumulated temperature after heading reached 1100 °C d. Approximately 3–5 mL of S7 or control solution was sprayed per pot, with application targeted to the panicle region. When the accumulated temperature after heading reached 1100 °C d, artificial rainfall was applied for four consecutive days to induce PHS. During the artificial rainfall treatment, the chamber temperature was maintained at a daily mean of 22 °C, with maximum and minimum temperatures of 27 °C and 17 °C, respectively, to reflect temperature conditions observed during the grain-filling period in the field [16]. Grains with visible radicle protrusion through the hull were considered germinated. PHS rate was calculated as the number of grains showing visible radicle protrusion divided by the total number of grains examined per replicate. The artificial rainfall treatment was conducted following a previously reported protocol for rice PHS induction [16].

To evaluate the effects of S7 on seed viability and root number, seeds were collected before artificial rainfall treatment when the accumulated temperature after heading reached 1100 °C d. Harvested seeds were subjected to accelerated ageing before germination assays, following a previously reported protocol for rice seeds [35]. Briefly, seeds were placed in sealed containers under high relative humidity (approximately 100%) and incubated at 45 °C for 24 h. After accelerated ageing, seeds were used to evaluate seed viability and root number under controlled conditions.

### Experiment 3: field trial

The field experiment was conducted in paddy fields at the National Institute of Crop Science, Jeonju, Republic of Korea. The PHS-susceptible floury rice cultivar ‘Baromi2’ was used to ensure sufficient PHS occurrence under natural field conditions and to evaluate the effectiveness of S7 in a susceptible genetic background [27].

Two field plots with different transplanting dates were used to obtain different heading dates within the same experimental year. Seedlings were transplanted on June 4 and June 14, 2024, and heading occurred on August 4 and August 10, respectively. S7 was applied on the same calendar date (September 4, 2024) in both plots. Because the plots differed in heading date and subsequent thermal accumulation after heading, the same S7 application date therefore corresponded to different intervals before the target accumulated-temperature stage. Based on the observed heading dates and accumulated temperature after heading, the S7 application corresponded to 8 and 14 d before the accumulated temperature reached 1100 °C d.

Rice panicles were sprayed with 250 μM S7 solution, whereas control panicles were sprayed with distilled water. The 250 μM concentration was selected because it effectively reduced PHS in the pot experiment while requiring a lower S7 input than the 500 μM treatment. PHS occurred under natural rainfall conditions during the field observation period. Following natural rainfall events, panicles were harvested on September 25, 2024, and PHS was evaluated based on grains showing visible radicle protrusion through the hull. Daily mean temperature and precipitation during the field observation period were recorded (Figure S2). PHS rate was calculated as the number of grains showing visible radicle protrusion divided by the total number of filled grains examined per replicate.

### S7 preparation

S7 was provided by Beom-Gi Kim of the Plant Biomaterials and Biotechnology Division, Department of Agricultural Biology, National Institute of Agricultural Sciences. A 100 mM stock solution of S7 was prepared by adding a wetting agent (EtOH 35%, polyethylene lauryl ether 45%, polyether-modified siloxane 5%, H₂O 15%) to S7. This stock solution was then diluted with distilled water to prepare S7 solutions at the required concentrations. For the pot experiment, approximately 3–5 mL of S7 solution was sprayed per pot, with application targeted to the panicle region. To evaluate whether the solvent/wetting-agent formulation itself affected seed germination, mock solutions corresponding to the 100 and 150 μM S7 treatments were prepared. These mock solutions contained the same final concentrations of the solvent/wetting-agent components used for the corresponding S7 treatments but did not contain S7, and were used as vehicle controls in an additional rice seed germination assay (Figure S3).

### Determination of α-amylase activity

Samples from the pot experiment were collected on days 1, 2, and 3 after artificial rainfall treatment, immediately frozen in liquid nitrogen, and stored at −80 °C. Each sample was ground into a fine powder for further analysis. For wheat, seed germination experimental materials were used. After treatment, the collected seeds were rapidly frozen in liquid nitrogen on days 0, 1, and 2 and ground. Thereafter, enzyme analysis was conducted using the same method as for rice. The α-amylase activity in whole rice and wheat samples was measured using Megazyme SD assay chemistry (K-AMYLSD) according to the K-AMYLSD Data booklet (04/2019; Megazyme) [25].

### RNA extraction and gene expression

Seeds were collected and immediately frozen in liquid nitrogen for RNA extraction. For each treatment, sixty rice seeds were used, and the samples were analyzed in triplicate for real-time polymerase chain reaction (PCR). Plant material for RNA extraction was collected at 3, 9, 18, and 24 h after S7 treatment and stored at − 80 °C. Total RNA was extracted following the description by Chang et al. [2]. cDNA was synthesized using a PrimeScript RT reagent kit with a gDNA eraser (Takara Bio Inc., Japan). For real-time PCR, SYBR Green (SYBR Real-time PCR Master Mix,Toyobo, Japan) was used as the fluorescent dye, and amplification was conducted using a Rotor-Gene 6000 (Corbett Research, Australia). Gene expression was quantified using quantitative real-time PCR using gene-specific primers. Primer sequences used in this study have been described previously [10, 29, 39] and are listed in Table S1. This procedure was performed as previously described [16].

### Extraction and measurement of ABA

The ABA extraction method was based on Guinn et al. [9], Choi et al. [4], and Lewis et al. [21]. Imbibed or germinating seeds (0.05 g) were frozen in liquid nitrogen and ground into a fine powder. The powder was extracted with 1 ml of 80% methanol and incubated overnight in a shaking incubator at 4 °C. The extract was centrifuged at 13,000 rpm for 10 min at 4 °C, and 500 µl of the supernatant was transferred to a 2 ml tube. For purification, 500 μl of 100% methanol was added to a C18 (Sep-Pak Vac) cartridge (Waters, USA), allowed to filter completely, and then 500 μl of the extract was added and filtered. After complete drainage, an additional 500 μl of 100% methanol was added and filtered again. The elute was concentrated using a SpeedVac at 30 °C for 3 h. Afterward, 500 μl of Tris-buffered saline solution, provided with the Phytodetek® ABA-ELISA test kit (Agdia Inc., Elkhart, IN, USA), was added to resuspend the samples. The suspension was centrifuged at 8000 rpm for 15 min at 4 °C, and the supernatant was transferred to a new 2 ml tube. ABA quantification was performed according to the manufacturer’s protocol for the Phytodetek® ABA-ELISA test kit. ABA content was expressed as pmol g⁻^1^ fresh weight.

### Statistical analysis

Statistical analyses were performed using SPSS version 26.0 (IBM Corp., Armonk, NY, USA) and R software version 4.3.1 (R Foundation for Statistical Computing, Vienna, Austria). Data are presented as means ± standard deviation (SD) or means ± standard error of the mean, as indicated in each figure legend. Comparisons among more than two treatments were analyzed using one-way analysis of variance, followed by Duncan’s multiple range test. Comparisons between two groups were performed using an independent-samples t-test. Statistical significance was set at *P* < 0.05.

## Results

To evaluate the potential role of S7 in suppressing PHS, we first examined its effects on rice seed germination, panicle-level PHS, α-amylase activity, and basic post-harvest seed performance. We then analyzed endogenous ABA content and ABA-related gene expression to determine whether the observed germination delay was associated with ABA accumulation or ABA signaling-related transcriptional responses.

### S7 delays early rice seed germination

**Fig. 1:**
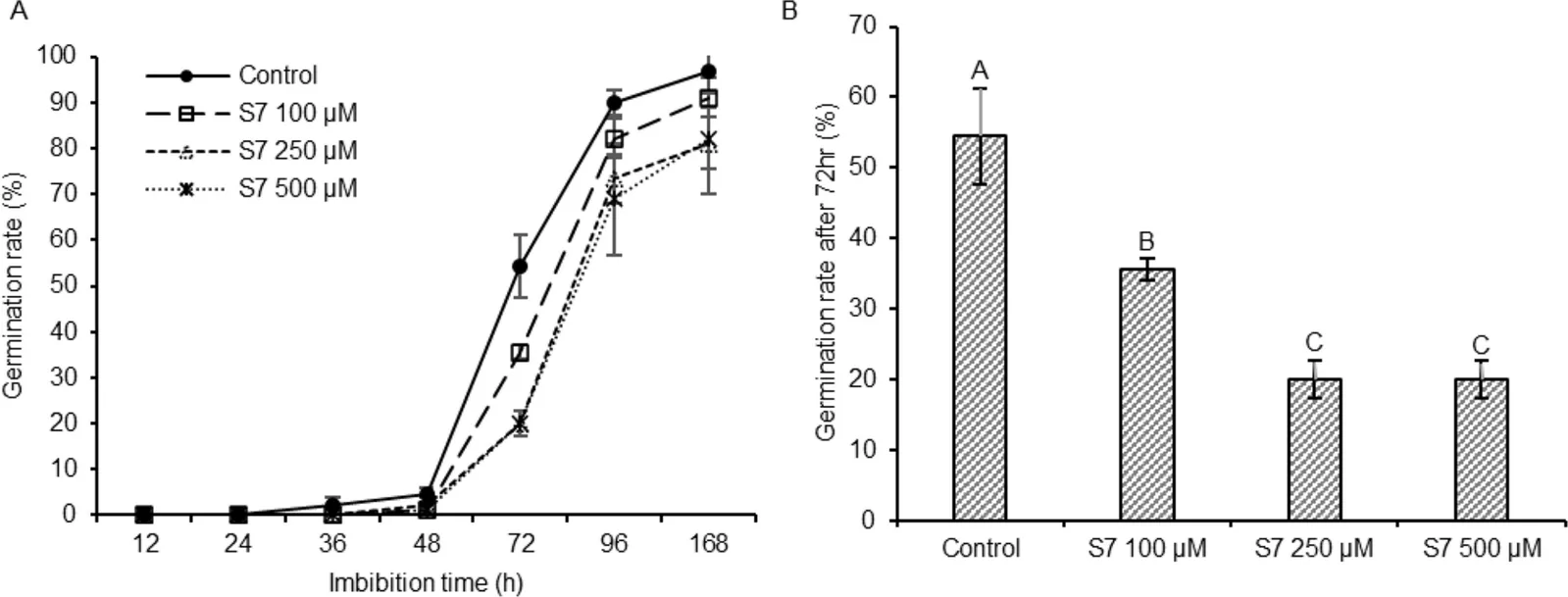
Effect of S7 concentration on rice seed germination. **A** Germination percentage of ‘Samkwang’ rice seeds grown on germination medium containing S7 at 0, 100, 250, or 500 μM. Germination was recorded at 12, 24, 36, 48, 72, 96, and 168 h after sowing. **B** Germination percentage at 72 h after sowing. Values represent means ± SD of three biological replicates, with 50 seeds per replicate. Different letters indicate significant differences among treatments at 72 h, as determined by one-way ANOVA followed by Duncan’s multiple range test (*P* < 0.05)

To determine whether S7 directly affects the onset and progression of seed germination, rice seeds were treated with different concentrations of S7, and germination was monitored over time. The effect of S7 on rice seed germination was evaluated at four concentrations: 0, 100, 250, and 500 μM. Germination was monitored at 12, 24, 36, 48, 72, 96, and 168 h after sowing (Fig. 1A). Germination was first observed in the control at 36 h, whereas visible germination in the S7-treated seeds was delayed until 48 h. At 72 h after sowing, S7 treatment markedly reduced germination in a concentration-dependent manner. The germination rate was approximately 35% lower in the 100 μM S7 treatment than in the control, whereas the 250 and 500 μM treatments reduced germination by approximately 63% compared with the control. At 96 h, the germination rates of the 100 and 250 μM S7 treatments were not significantly different from that of the control, whereas the 500 μM treatment still showed delayed germination. By 168 h, germination rates had largely recovered in all S7 treatments, with no significant differences among treatments.

Because the inhibitory effect of S7 was most pronounced at 72 h, germination rates at this time point were further compared (Fig. 1B). All S7 treatments significantly reduced early seed germination compared with the control, and the inhibitory effect increased with S7 concentration. These results indicate that S7 delays early seed germination in rice in a concentration-dependent manner, but this effect is largely transient rather than permanent.

To determine whether the solvent/wetting-agent formulation contributed to the germination-delaying effect, an additional germination assay was conducted using mock solutions corresponding to the 100 and 150 μM S7 treatments. Although some formulation-related effects were observed, S7 treatments showed a stronger delay in early germination and a greater reduction in α-amylase activity than the corresponding mock treatments (Figure S3). These results suggest that the germination-delaying response observed in S7-treated seeds cannot be explained solely by the solvent/wetting-agent formulation.

### S7 treatment timing and concentration affect panicle-level PHS suppression under controlled-environment conditions

To determine how S7 concentration and application timing affect panicle-level PHS suppression under controlled-environment conditions, rice panicles were treated with S7 at 250 or 500 μM at 3, 7, or 10 d before the accumulated temperature after heading reached 1100 °C d, followed by artificial rainfall treatment. α-Amylase activity was measured in parallel to examine whether changes in PHS were associated with altered starch-degradation activity during germination.

**Fig. 2:**
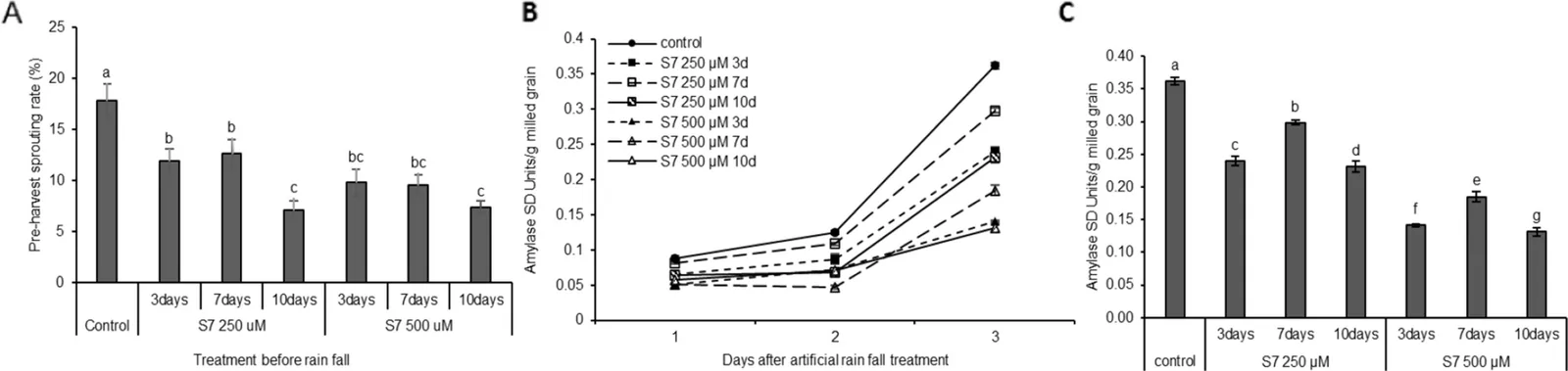
Effects of S7 on PHS and α-amylase activity under artificial rainfall. **A** PHS rate after 96 h of artificial rainfall. Control solution or S7 solutions at 250 or 500 μM were sprayed onto the panicle region of rice plants at approximately 3–5 mL per pot at 3, 7, or 10 d before the accumulated temperature after heading reached 1100 °C d. **B** Time-course changes in α-amylase activity in rice grains during artificial rainfall treatment. **C** α-Amylase activity in rice grains on day 3 after the start of artificial rainfall treatment. Values represent means ± SEM (*n* = 3). Different letters in panels A and C indicate significant differences among treatments, as determined by one-way ANOVA followed by Duncan’s multiple range test (*P* < 0.05)

In the control, the PHS rate was 17.79% after 4 d of artificial rainfall (Fig. 2A). S7 treatment significantly reduced PHS, and the extent of suppression differed among application timing and concentration combinations. At 250 μM, the PHS rates were 11.93%, 12.60%, and 7.09% when S7 was applied 3, 7, and 10 d before artificial rainfall, respectively, corresponding to reductions of approximately 33%, 29%, and 60% compared with the control. At 500 μM, the PHS rates were reduced to 9.78%, 9.57%, and 7.33%, corresponding to reductions of approximately 45%, 46%, and 59%, respectively. The reduction in PHS was most pronounced in the 10 d pre-treatment groups under the pot experimental conditions. These results indicate that S7-mediated suppression of panicle-level PHS varied among treatment combinations, with the 10 d pre-treatment showing the greatest suppression under the present controlled-environment conditions.

Changes in α-amylase activity after artificial rainfall were generally consistent with the PHS response (Fig. 2B). α-Amylase activity increased over time after rainfall treatment but remained lower in the S7-treated groups than in the control. On day 3 after artificial rainfall, α-amylase activity was highest in the control and was lower in the S7-treated groups, with the lowest values observed in the 10 d pre-treatment groups (Fig. 2C). Among the S7 treatment combinations, the lowest α-amylase activity was observed in the 500 μM treatment applied 10 d before artificial rainfall. These results suggest that S7-mediated suppression of panicle-level PHS under simulated rainfall conditions was associated with reduced α-amylase activity during early germination.

### S7 treatment does not impair seed viability or root number under the tested conditions

**Fig. 3:**
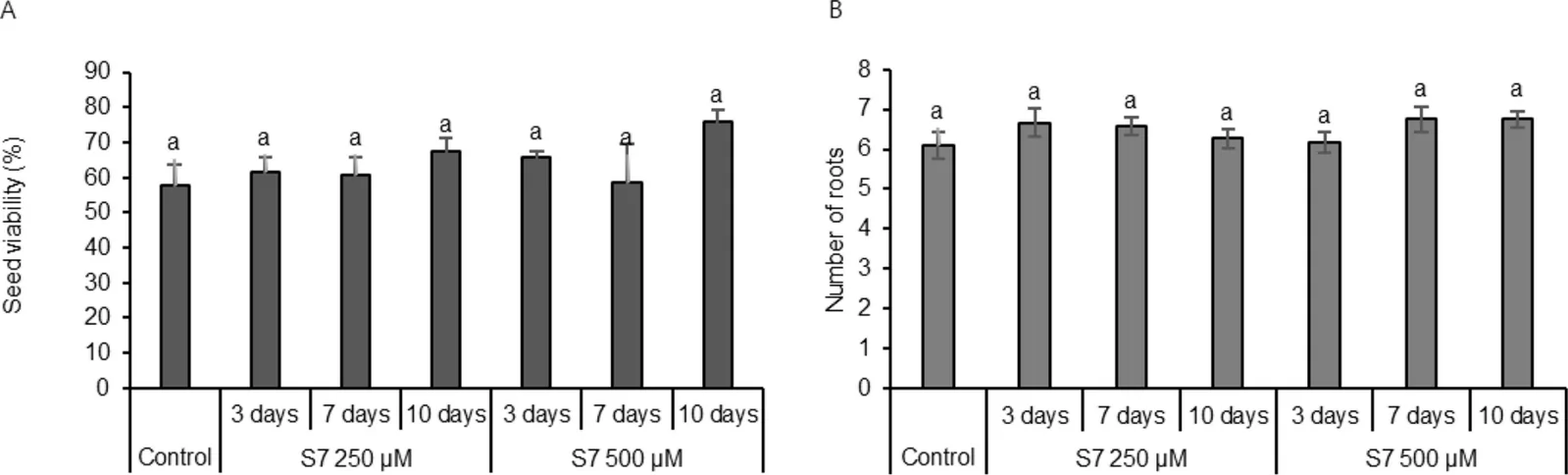
Effect of S7 treatment on seed viability and root number after accelerated ageing. Control solution or S7 solutions at 250 or 500 μM were sprayed onto the panicle region of rice plants at approximately 3–5 mL per pot at 3, 7, or 10 d before the accumulated temperature after heading reached 1100 °C d. Seeds were collected when the accumulated temperature after heading reached 1100 °C d, subjected to accelerated ageing treatment, and then used to evaluate seed viability and root number. **A** Seed viability. **B** Number of roots. Values represent means ± SEM of 20 seeds with three independent replicates. Statistical differences among treatments were determined using one-way ANOVA followed by Duncan’s multiple range test (*P* < 0.05). No significant differences were observed among treatments

Because a PHS-suppressive treatment should not compromise basic post-harvest seed performance, seed viability and root number were evaluated using seeds collected from the pot experiment before artificial rainfall treatment (Fig. 3). Seeds were harvested when the accumulated temperature after heading reached 1100 °C d and subjected to accelerated ageing before germination and seedling evaluation.

Seed viability did not differ significantly between the control and S7-treated groups, regardless of S7 concentration or application timing (Fig. 3A). Similarly, no significant differences in root number were observed among treatments (Fig. 3B). These results indicate that S7 treatment did not significantly affect seed viability or root number under the tested conditions.

### S7 treatment reduces PHS under the tested field conditions

**Fig. 4:**
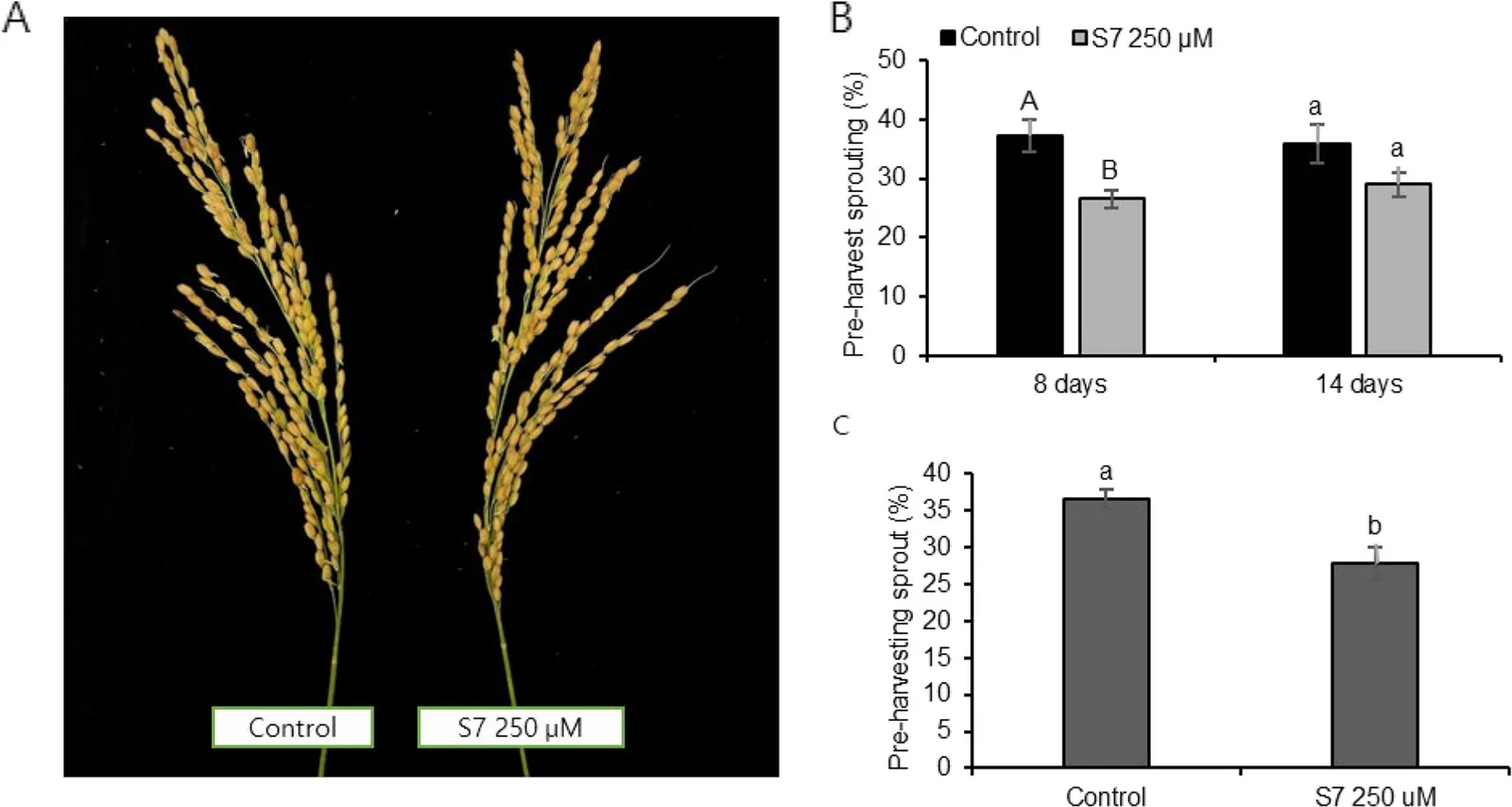
Effect of S7 treatment on PHS under field conditions with different transplanting dates. **A** Representative rice panicles showing PHS under natural rainfall conditions. **B** PHS rate under two field conditions with different transplanting dates and heading dates. **C** Mean PHS rate averaged across the two field conditions. Rice panicles of the PHS-susceptible cultivar ‘Baromi2’ were sprayed with 250 μM S7; control panicles received distilled water. Because heading dates differed between the two transplanting-date conditions, the same calendar-date S7 application corresponded to 8 and 14 d before the accumulated temperature after heading reached 1100 °C d. PHS was evaluated under natural rainfall conditions. Data are presented as means ± SEM (*n* = 3). Different letters in panel B indicate significant differences among treatments according to one-way analysis of variance followed by Duncan’s multiple range test (*P* < 0.05). The difference between control and S7 treatment in panel C was assessed using an independent-samples t-test (*P* < 0.05)

To examine whether the PHS-suppressive effect observed under controlled-environment conditions could also be detected under natural rainfall conditions, rice panicles of the PHS-susceptible cultivar ‘Baromi2’ were sprayed with 250 μM S7 during grain filling (Fig. 4). Two field conditions with different transplanting dates were used to evaluate the field performance of S7 under different heading dates and grain-filling environments. Because heading date and thermal accumulation differed between the two transplanting-date conditions, the same calendar-date S7 application corresponded to 14 and 8 d before the accumulated temperature after heading reached 1100 °C d. PHS was assessed after naturally occurring rainfall events, and daily mean temperature and precipitation were recorded during the field observation period (Figure S2).

When S7 application corresponded to 8 d before the accumulated temperature reached 1100 °C d, the PHS rate decreased significantly from 37.2% in the control to 26.6% in the S7-treated group (Fig. 4B). When S7 application corresponded to 14 d before this stage, the PHS rate decreased from 35.8% in the control to 29.0% in the S7-treated group; however, this reduction was not statistically significant. When data from the two field conditions were combined, the mean PHS rate was significantly lower in the S7-treated group than in the control, decreasing from 36.5% to 27.8% (Fig. 4C). This combined comparison was used to summarize the overall tendency of S7-treated panicles to show lower PHS under field conditions, whereas the two field conditions were also interpreted separately because they differed in heading date and thermal accumulation pattern.

These results indicate that S7 treatment reduced PHS under the tested field conditions. However, the magnitude of suppression varied between the two field conditions with different heading dates and thermal accumulation patterns. The clearer suppressive response observed in the 8 d group was consistent with the pot experiment, in which S7 was effective when applied approximately 7–10 d before the target accumulated temperature stage. Therefore, the field results should be interpreted as supporting the field applicability of S7 under variable phenological and grain-filling conditions, rather than as a fully controlled comparison of application timing.

### S7 does not increase endogenous ABA accumulation during seed imbibition

**Fig. 5:**
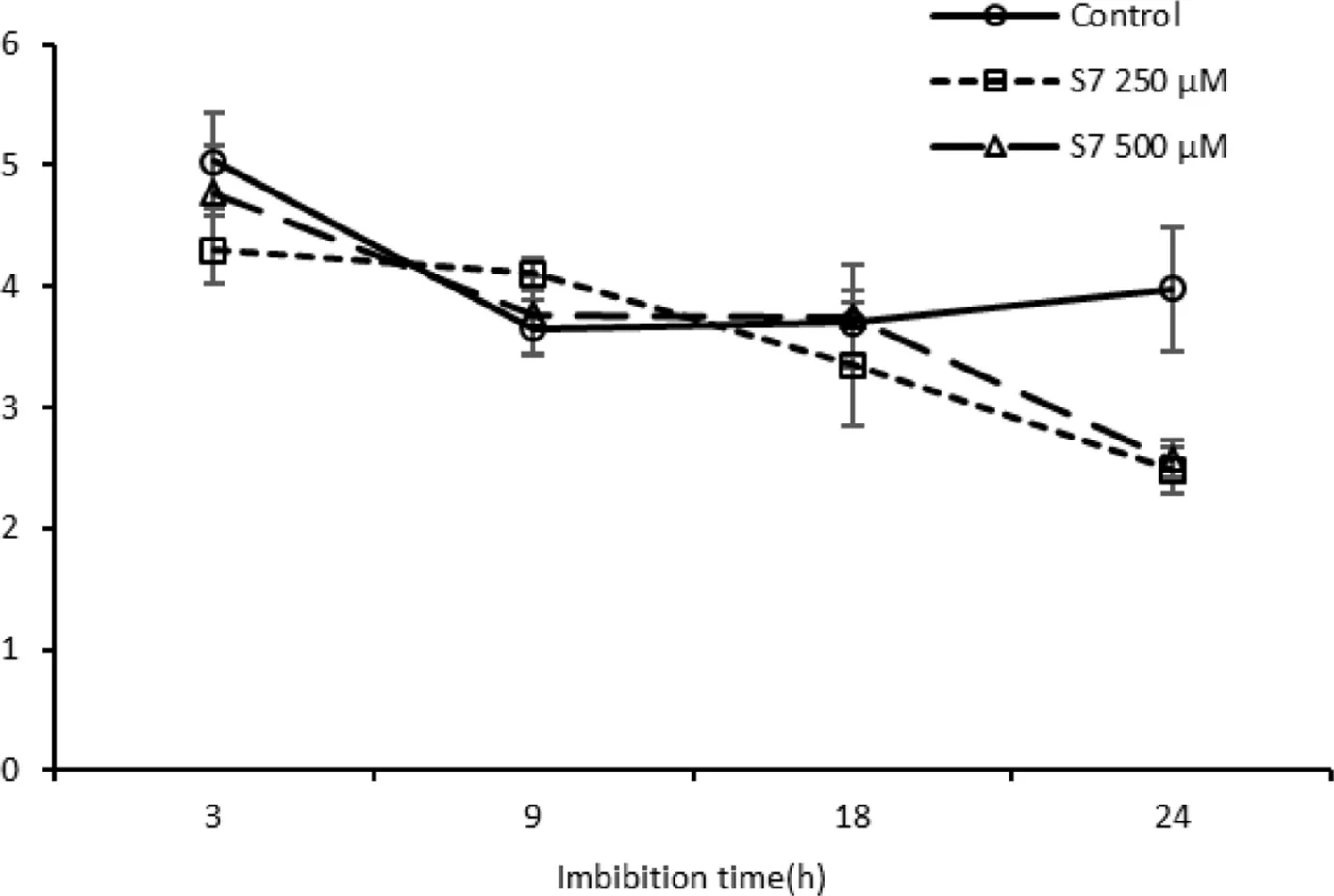
Effect of S7 on endogenous ABA content during rice seed imbibition. Rice seeds were soaked in water as the control or in S7 solutions at 250 or 500 μM. Endogenous ABA content was quantified at 3, 9, 18, and 24 h after imbibition. Values represent means ± SEM (*n* = 3 biological replicates)

To determine whether S7-mediated germination delay was associated with increased endogenous ABA accumulation, ABA levels were measured in seeds soaked in water or in S7 solutions at 250 or 500 μM. Samples were collected at 3, 9, 18, and 24 h after imbibition (Fig. 5). In the control, ABA levels were 5.03, 3.65, 3.70, and 3.98 pmol g⁻^1^ fresh weight at 3, 9, 18, and 24 h, respectively, showing an initial decrease from 3 to 9 h, followed by relatively stable levels thereafter. In the 250 μM S7 treatment, ABA levels were 4.30, 4.10, 4.36, and 2.48 pmol g⁻^1^ fresh weight at the corresponding time points, whereas in the 500 μM S7 treatment, they were 4.77, 3.77, 3.74, and 2.57 pmol g⁻^1^ fresh weight, respectively.

During the first 18 h of imbibition, ABA levels in S7-treated seeds remained comparable to those in the control. At 24 h, ABA levels tended to be lower in both S7 treatments than in the control. No concentration-dependent increase in ABA level was observed in response to S7 treatment. These results indicate that S7-mediated germination delay was not accompanied by increased endogenous ABA accumulation during seed imbibition, suggesting that its inhibitory effect is unlikely to be driven by enhanced ABA biosynthesis or accumulation.

### S7 treatment is associated with ABA signaling-related transcriptional responses during seed imbibition

**Fig. 6:**
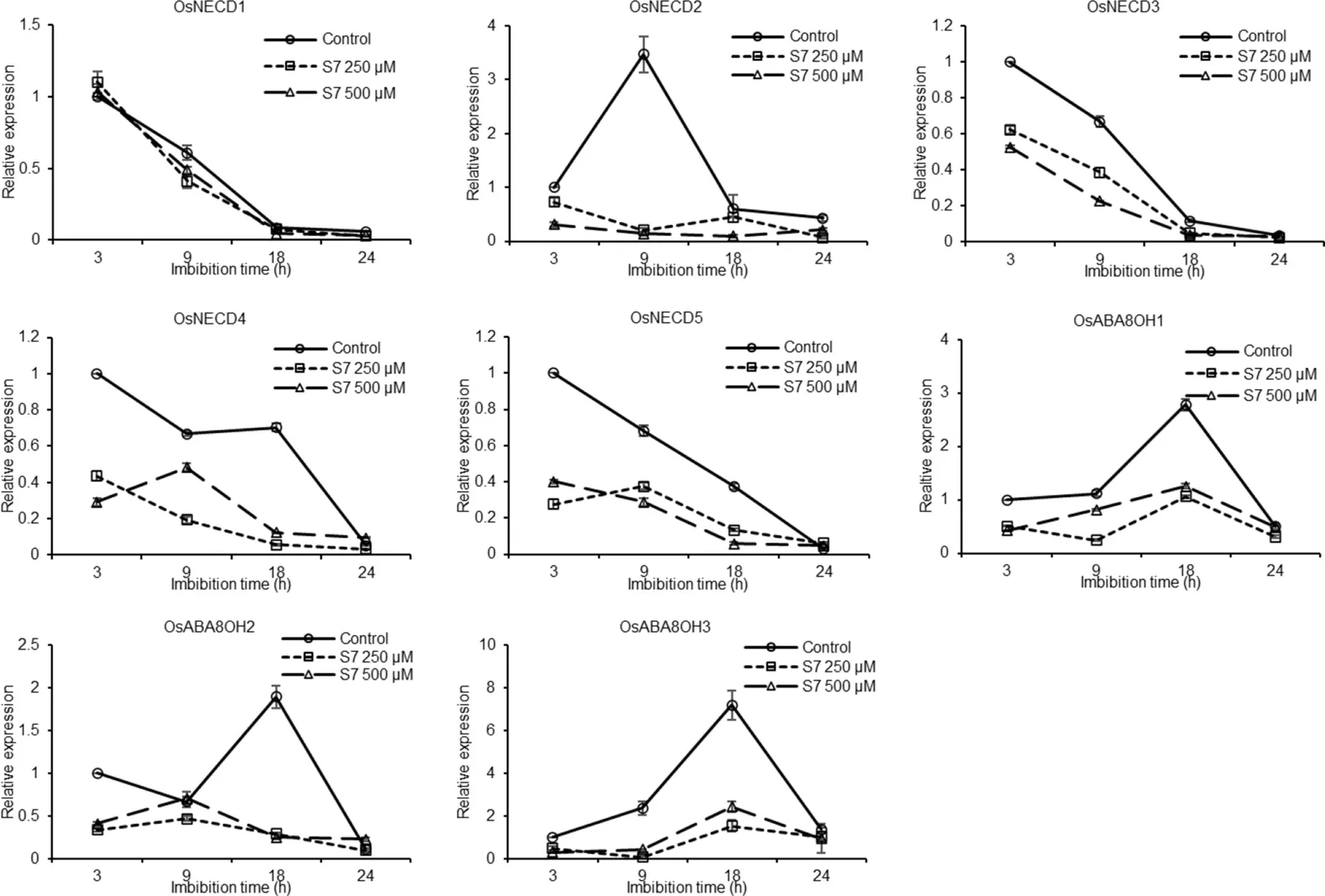
Effects of S7 on ABA metabolism-related gene expression. Relative expression levels of ABA biosynthesis-related genes (*OsNCED1*, *OsNCED2*, *OsNCED3*, *OsNCED4*, and *OsNCED5*) and ABA catabolism-related genes (*OsABA8OH1*, *OsABA8OH2*, and *OsABA8OH3*) in rice seeds treated with S7 at 0, 250, or 500 μM. Gene expression levels were normalized to *OsUBQ5*. Values represent means ± SD (*n* = 3 biological replicates)

Because S7-mediated germination delay was not accompanied by increased endogenous ABA accumulation, we next examined whether S7 treatment was associated with changes in ABA metabolism- and signaling-related gene expression during early seed imbibition. Transcript levels of ABA biosynthetic genes, including OsNCED1, OsNCED2, OsNCED3, OsNCED4, and OsNCED5, and ABA catabolic genes, including OsABA8OH1, OsABA8OH2, and OsABA8OH3, generally decreased in S7-treated seeds compared with the control, regardless of S7 concentration (Fig. 6). This expression pattern was consistent with the endogenous ABA measurements, supporting the observation that S7 treatment did not promote ABA accumulation during seed imbibition.

**Fig. 7:**
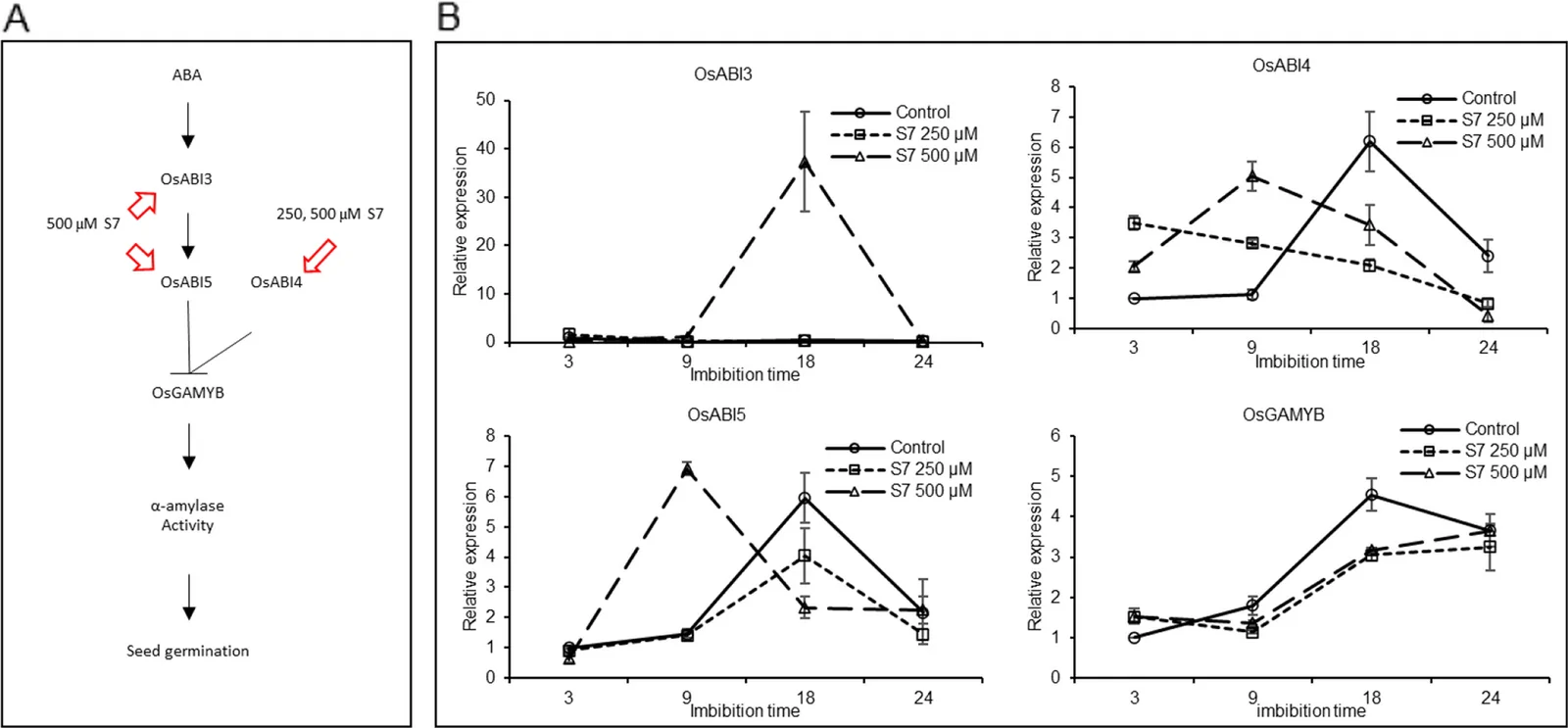
Effect of S7 on ABA signaling-related and downstream gene expression during rice seed imbibition. **A** Literature-supported working model used to contextualize ABA signaling-related transcriptional responses and downstream germination-associated outputs during rice seed imbibition. The model integrates the reported ABA signaling–GAMYB–hydrolytic enzyme framework with the S7-associated responses observed in this study. Red labels indicate genes or physiological outputs measured in this study that showed S7-associated changes. The arrows in this model indicate literature-supported regulatory relationships and S7-associated changes observed in this study; they do not indicate experimentally validated direct regulation by S7. **B** Relative expression levels of ABA signaling-related genes (*OsABI3*, *OsABI4*, and *OsABI5*) and the downstream germination-related gene *OsGAMYB* in rice seeds treated with S7 at 0, 250, or 500 μM. Gene expression levels were normalized to *OsUBQ5*. Values represent means ± SD (*n* = 3 biological replicates)

In contrast, S7 treatment affected the expression of ABA signaling-related genes (Fig. 7). At the higher S7 concentration of 500 μM, OsABI3 and OsABI5 were induced earlier than in the control, suggesting earlier activation of ABA signaling-related transcriptional responses during imbibition. Notably, OsABI4 showed a more consistent response to S7 treatment, with elevated transcript levels at earlier time points in both the 250 and 500 μM S7 treatments compared with the control. This early induction suggests that OsABI4 may be particularly responsive to S7 treatment during early imbibition.

Consistent with the induction of ABA signaling-related genes, the transcript level of OsGAMYB, a positive regulator of α-amylase expression, was reduced in S7-treated seeds compared with the control (Fig. 7). This reduction in OsGAMYB expression was consistent with the lower α-amylase activity observed in S7-treated seeds. Together, these results suggest that S7-mediated germination delay and PHS suppression were associated with ABA signaling-related transcriptional changes, including early induction of OsABI4 and reduced OsGAMYB expression, without promoting ABA biosynthesis or accumulation.

### Additional wheat assay for S7-mediated germination delay

To examine whether the germination-delaying effect of S7 could also be detected in another cereal crop, wheat seeds were treated with S7 at 150, 300, and 450 μM (Figure S4). S7 delayed early wheat seed germination in a concentration-dependent manner, with the strongest inhibition observed at 36 h after sowing. The time to 50% germination increased from 31.7 h in the control to 43.4, 46.5, and 53.4 h in the 150, 300, and 450 μM S7 treatments, respectively. S7 treatment also reduced α-amylase activity in wheat seeds. These results indicate that S7 delayed early germination in wheat under the tested conditions, in association with reduced α-amylase activity.

## Discussion

This study showed that S7 delayed early seed germination and reduced PHS in rice under controlled-environment and field conditions, without increasing endogenous ABA accumulation during seed imbibition. S7 treatment delayed early germination (Fig. 1), reduced α-amylase activity and panicle-level PHS under simulated rainfall conditions in the pot experiment (Fig. 2), and reduced PHS under the tested field conditions (Fig. 4). In contrast, endogenous ABA levels did not increase following S7 treatment during seed imbibition (Fig. 5). These findings indicate that the inhibitory effect of S7 on germination and PHS is unlikely to be primarily driven by enhanced ABA biosynthesis or accumulation. Instead, the results support a sequential interpretation in which S7-mediated germination delay is associated with reduced α-amylase activity and PHS suppression, while the accompanying molecular response appears to involve ABA signaling-related transcriptional changes rather than increased ABA accumulation.

Several chemical approaches for suppressing PHS have been linked to ABA metabolism, α-amylase activity, and starch degradation. For example, eugenol has been reported to reduce PHS in rice by increasing ABA content, modulating ABA metabolism-related gene expression, and suppressing α-amylase activity [10]. Similarly, combined treatment with maleic hydrazide and coumarin suppresses rice seed germination by altering reactive oxygen species accumulation, ABA metabolism, and starch degradation [34]. These studies are similar to the present study in that chemical treatment reduced premature germination and germination-associated α-amylase activity. However, they largely interpreted PHS suppression in relation to increased ABA accumulation, altered ABA metabolism, reactive oxygen species regulation, or downstream inhibition of starch degradation. In contrast, S7 did not increase endogenous ABA levels during seed imbibition in the present study (Fig. 5). Moreover, transcript levels of ABA biosynthesis- and catabolism-related genes, including *OsNCED1–OsNCED5* and *OsABA8OH1–OsABA8OH3*, generally decreased in S7-treated seeds (Fig. 6). Together with the ABA quantification results, these expression patterns support the interpretation that S7 delayed germination without promoting ABA accumulation. Thus, S7 may complement previously reported chemical PHS-control approaches by providing a synthetic partial ABA agonist-based approach that is associated with germination suppression without increased endogenous ABA accumulation, although further functional validation is required to clarify the precise signaling mechanism.

Although S7 did not increase endogenous ABA accumulation, it was associated with changes in ABA signaling-related gene expression during early seed imbibition (Fig. 7). In particular, S7 treatment was accompanied by earlier induction of *OsABI4* and reduced expression of *OsGAMYB* (Fig. 7). ABI transcription factors, including ABI3, ABI4, and ABI5, are key regulators of seed dormancy and germination, and ABA signaling is known to antagonize gibberellin (GA)-responsive germination processes, including α-amylase induction [36]. In rice, Park et al. [29] reported that altered ABA signaling in the osdsg1 mutant was accompanied by increased expression of ABA signaling-related genes and reduced expression of *OsGAMYB* and downstream genes encoding hydrolytic enzymes. They further proposed a working model in which OsABI4 and OsABI5 act as negative regulators of *OsGAMYB*, whereas OsGAMYB positively regulates hydrolytic enzyme genes required for germination. This previous finding supports a link between ABA signaling status and repression of germination-associated hydrolytic activity. In cereals, GAMYB functions as a positive regulator of α-amylase expression during germination [8], [13]. Therefore, the reduced *OsGAMYB* expression observed in S7-treated seeds is consistent with the lower α-amylase activity and delayed germination observed in this study (Figs. 1, 2, and 7). Within this previously proposed ABA signaling–GAMYB–hydrolytic enzyme framework, the early induction of *OsABI4* and the reduced expression of *OsGAMYB* in S7-treated seeds provide a pathway-consistent explanation for the reduced α-amylase activity and delayed germination observed in this study. However, the proposed link between ABA signaling-related transcriptional responses and reduced α-amylase activity is based on correlative gene expression and enzyme activity data. Therefore, this relationship should be interpreted as an associated response, rather than direct evidence for pathway control. Future studies should test this relationship more directly by examining whether exogenous GA₃ can rescue S7-mediated germination delay and α-amylase suppression and by evaluating S7 responses in functional lines, such as *OsGAMYB*-overexpression or *OsABI4* loss-of-function plants.

This interpretation is consistent with previous work showing that S7 functions as a synthetic partial ABA agonist. Min et al. [26] reported that S7 selectively mediates interactions between specific OsPYL receptors and OsPP2CA phosphatases and induces only a subset of ABA-responsive transcriptional outputs, without fully mimicking all physiological effects of ABA. More recently, Song et al. [37] showed that engineered OsPYL5 receptor variants enhanced ABA signaling and delayed germination, indicating that manipulation of ABA receptor signaling can reinforce germination-repressive responses in rice. Together, these studies suggest that ABA signaling in rice can be modulated through either chemical agonism or receptor engineering. The present study extends this concept to PHS-related conditions by showing that S7 delayed germination and reduced PHS without increasing endogenous ABA accumulation (Figs. 1, 2, and 5). Thus, S7 may provide a useful chemical approach for suppressing germination through ABA signaling-related responses without direct ABA application or genetic modification. This interpretation is also broadly consistent with previous reports showing that enhanced ABA signaling is associated with stronger seed dormancy and PHS resistance in rice [15]. Taken together, these findings suggest that chemical modulation of ABA receptor-mediated signaling may help reinforce germination-repressive responses during the period when mature grains are vulnerable to PHS.

The controlled-environment pot experiment extended the germination-delaying effect of S7 to panicle-level PHS suppression under simulated rainfall conditions. The pot experiment showed that the PHS-suppressive effect of S7 differed depending on the combination of treatment timing and concentration (Fig. 2). At 3 and 7 d before the target accumulated-temperature stage, the 500 μM treatment tended to show lower PHS rates than the 250 μM treatment. However, when S7 was applied 10 d before the accumulated temperature after heading reached 1100 °C d, both the 250 and 500 μM treatments showed similarly low PHS rates (Fig. 2). These results suggest that the response to S7 may vary depending on treatment timing and concentration under controlled-environment conditions, and that 250 μM may be sufficient under specific treatment conditions. The concomitant reduction in α-amylase activity further supports an association between panicle-level PHS suppression and reduced germination-associated starch-degradation activity. In addition, S7 did not significantly reduce seed viability or root number after accelerated ageing under the tested conditions (Fig. 3), suggesting that these treatment conditions did not impair basic indicators of post-harvest seed performance. Nevertheless, further evaluation of seedling establishment and field emergence is necessary before practical application.

The field trial provided field-level support for the potential use of S7 in PHS management under agronomically relevant conditions. In the PHS-susceptible cultivar ‘Baromi2’, 250 μM S7 reduced PHS under the tested natural rainfall conditions (Fig. 4). Because heading date and thermal accumulation differed between the two transplanting-date conditions, the same calendar-date application corresponded to 8 and 14 d before the accumulated temperature after heading reached 1100 °C d. Although the two field conditions differed in response magnitude, the combined analysis showed a lower mean PHS rate in S7-treated panicles than in the control (Fig. 4C). Because PHS occurrence in rice is strongly influenced by weather conditions during grain filling, including rainfall and temperature before harvest [16], the field response observed under natural rainfall likely reflects more variable but agronomically realistic environmental conditions (Figure S2). These results suggest that S7 may reduce PHS under field conditions, although the magnitude of suppression may vary with phenological stage, grain-filling environment, and application timing relative to grain maturation. Therefore, further field trials across years, transplanting dates, cultivars, and locations are needed to refine the optimal application window and field-use conditions.

Because the rice germination assay, α-amylase activity measurement, ABA quantification, and gene expression analyses were conducted using ‘Samkwang’, the physiological and ABA-related transcriptional responses observed in this study should be interpreted primarily in the context of this cultivar. Although the field experiment using the PHS-susceptible cultivar ‘Baromi2’ suggests that S7 may reduce PHS under natural rainfall conditions in a susceptible cultivar, the present study did not directly compare S7 responses among rice genotypes with contrasting dormancy levels or PHS susceptibility under identical laboratory and pot conditions. Therefore, further validation using genetically diverse rice cultivars will be required before the efficacy and ABA-related responses of S7 can be broadly generalized across rice germplasm.

Finally, after evaluating the effect of S7 in rice, the wheat germination assay was included as an additional seed-level cereal assay to examine whether S7-mediated germination delay could also be detected in another cereal crop under the tested conditions. S7 delayed early germination and reduced α-amylase activity in wheat (Figure S4), but the wheat assay was not designed to compare rice and wheat sensitivity because different concentration ranges were used. Moreover, ABA content and ABA signaling-related gene expression were not examined in wheat. Therefore, the wheat result should be interpreted only as an additional seed-level cereal assay, rather than as evidence for a direct rice–wheat sensitivity comparison or a conserved molecular mechanism across cereal crops.

Overall, this study indicates that S7 reduces PHS in rice in association with delayed early germination, reduced α-amylase activity, and ABA signaling-related transcriptional responses, rather than increased endogenous ABA accumulation (Figs. 1, 2, and 5–7). The combined results support a working model in which S7-mediated ABA signaling-related responses are associated with repression of *OsGAMYB*, reduced α-amylase activity, delayed germination, and reduced PHS under controlled-environment and field conditions. These findings highlight the potential of S7 as a non-genetic chemical tool for PHS management in rice. Further studies are required to optimize concentration, application timing, formulation, environmental stability, and cultivar-specific responses under field conditions.

## Conclusions

This study showed that S7 delayed early seed germination and reduced PHS in rice in association with reduced α-amylase activity, without increasing endogenous ABA accumulation during seed imbibition. S7 treatment did not promote the expression of ABA biosynthesis-related genes; instead, it was associated with ABA signaling-related transcriptional responses, including early induction of *OsABI4* and repression of *OsGAMYB*. These results suggest that S7-mediated suppression of germination is associated with ABA signaling-related transcriptional responses and reduced α-amylase activity, rather than increased ABA accumulation. Under controlled-environment conditions, the suppressive effect of S7 was influenced by application timing relative to grain maturation and accumulated temperature after heading. Field evaluation further supported the potential applicability of S7 under variable phenological and grain-filling conditions. Overall, S7 may serve as a potential chemical tool for reducing pre-harvest sprouting in rice, although further studies are required to optimize concentration, application timing, formulation, environmental stability, and cultivar-specific responses under field conditions.

## Supplementary Information

Supplementary Material 1.

## Data Availability

The data supporting the findings of this study are available from the corresponding authors upon reasonable request.

## Abbreviations

ABA: Abscisic acid
ABA-ELISA: Abscisic acid enzyme-linked immunosorbent assay
GA: Gibberellin
MeJA: Methyl jasmonate
*OsABA8OH*: ABA 8′-hydroxylase gene family in rice
*OsABI*: ABA INSENSITIVE gene family in rice
*OsGAMYB*: Gibberellin-inducible MYB transcription factor in rice
*OsNCED*: 9-Cis-epoxycarotenoid dioxygenase gene family in rice
PCR: Polymerase chain reaction
PHS: Pre-harvest sprouting
qPCR: Quantitative polymerase chain reaction
SEM: Standard error of the mean
SD: Standard deviation
°C d: Degree days
