# Supplementary material for: Evaluation of S7, a synthetic partial abscisic acid agonist, as a potential suppressor of pre-harvest sprouting in rice
Source: BMC Plant Biol. 2026 Jul 16;26:1737. doi: 10.1186/s12870-026-09511-9 (PMC13651045; doi:10.1186/s12870-026-09511-9)
Supplement: Supplementary file 1 [file 12870_2026_9511_MOESM1_ESM.docx]

**Figure S1. Controlled-environment facility and artificial rainfall system used in this study.** (A) Controlled-environment facility at the National Institute of Crop Science, Jeonju, Republic of Korea. (B) Lighting chamber in which light intensity, temperature, photoperiod, and relative humidity could be regulated. (C) Artificial rainfall treatment system used to induce pre-harvest sprouting (PHS) in rice panicles under controlled conditions.

**Figure S2. Daily mean temperature and precipitation during the grain-filling period under two transplanting dates.** The first and second transplanting were conducted on 4 June and 14 June 2024, respectively. Arrows indicate key phenological and experimental events, including heading date, S7 treatment application, the timing at which accumulated temperature after heading reached 1100 °C d, and PHS assessment. T1 and T2 denote the first and second transplanting dates, respectively. Yellow and green arrows correspond to T1 and T2, respectively, whereas the red arrow indicates S7 treatment application. Because heading occurred on different calendar dates between transplanting dates, the 1100 °C d threshold was reached on different dates. After naturally occurring rainfall events during the grain-filling period, panicles were harvested on 25 September 2024 and evaluated for PHS.

**Figure S3. Effects of S7 and corresponding mock treatments on seed germination and α-amylase activity in rice.** (A) Rice seeds were imbibed in distilled water as the control, mock solutions corresponding to the 100 and 150 µM S7 treatments, or S7 solutions at 100 and 150 µM. Germination rates were recorded at the indicated imbibition times (12, 18, 24, 36, 48, 60, and 168 h). The mock solutions contained the same final concentrations of the solvent/wetting-agent components used for the corresponding S7 treatments but did not contain S7. (B) Germination percentage at 72 h after sowing. (C) Effects of S7 and corresponding mock treatments on α-amylase activity (SD U g⁻¹ milled grain). (D) α-amylase activity in rice grains on day 2 after imbibition. Data are presented as means ± SD of three biological replicates, with 50 seeds per replicate. Statistical differences among treatments were assessed using one-way analysis of variance followed by Duncan’s multiple range test; bars labeled with different letters are significantly different at P < 0.05.

**Figure S4. Effect of S7 concentration on wheat seed germination and α-amylase activity.** (A) Representative images of wheat seeds 4 d after sowing. (B) Germination percentage of wheat seeds treated with S7 at 0, 150, 300, or 450 μM. Germination was recorded at 12, 18, 24, 36, 48, 60, 72, and 84 h after sowing. (C) α-Amylase activity in wheat seeds treated with different S7 concentrations. Values represent means ± SD (n = 3 biological replicates, 50 seeds per replicate for germination assay). Different letters indicate significant differences among treatments, as determined by one-way analysis of variance followed by Duncan’s multiple range test (P < 0.05).

**Table S1. Real-time PCR primer list**

| No. | oligo name | 5' - Oligo Seq - 3' |
| --- | --- | --- |
| 1 | OsUBQ5-f1 | 5' CCCTTGCCGACTATAACATCC 3' |
| 2 | OsUBQ5-r1 | 5' CAACACAACCATGACACGATG 3' |
| 3 | OsABI5-f1 | 5'-ATGGCATCGGAGATGAGCAAGAAC-3' |
| 4 | OsABI5-r1 | 5'-GCTTCTTTGTCAGTAGAACCGTCTTC-3' |
| 5 | OsABI3-f1 | 5'-CCCAACAACAAAAGCAGGAT-3' |
| 6 | OsABI3-r1 | 5'-CCTTTGTATTGGACGAGACG-3' |
| 7 | OsABI4-f1 | 5'-TTCCATCACCAACCGTTC-3' |
| 8 | OsABI4-r1 | 5'-TTGAGGAAGAGATCGAACCA-3' |
| 9 | OsGAMYB-f1 | 5'-GAATCCACCCCTCCTGTT-3' |
| 10 | OsGAMYB-r1 | 5'-GCCCCATTACTTGCTCTC-3' |
| 11 | OsNCED1-f1 | 5' AGCCTCGGTCTTCCAATTTT 3' |
| 12 | OsNCED1-r1 | 5' CACCCAACACAAAAGCTACG 3' |
| 13 | OsNCED2-f1 | 5' GGTATGGAAACGAGGATAGTGGTT 3' |
| 14 | OsNCED2-r1 | 5' TGCTTATTGTTGTGCGAGAAGTTC 3' |
| 15 | OsNCED3-f1 | 5' AACCATCCAAACCGACGA 3' |
| 16 | OsNCED3-r1 | 5' TGCCGAGAGAGAGAGAGTGTG 3' |
| 17 | OsNCED4-f1 | 5' AACCATCCAAACCGACGA 3' |
| 18 | OsNCED4-r1 | 5' TGCCGAGAGAGAGAGAGTGTG 3' |
| 19 | OsNCED5-f1 | 5' CGCCATCACCGAGAACTT 3' |
| 20 | OsNCED5-r1 | 5' CTCGCCGAGCTTGAACAC 3' |
| 21 | OsABA8OH1-f1 | 5' TCAACACCTTCCAAGAGATGAA 3' |
| 22 | OsABA8OH1-r1 | 5' ATCTCCTCCTCCCCGAAG 3' |
| 23 | OsABA8OH2-f1 | 5' CTACTGCTGATGGTGGCTGA 3' |
| 24 | OsABA8OH2-r1 | 5' CCCATGGCCTTTGCTTTAT 3' |
| 25 | OsABA8OH3-f1 | 5' TGGGTTGGCCTTACATCG 3' |
| 26 | OsABA8OH3-r1 | 5' TGAAGATCTCGCCGTACCTC 3' |
